# Pre-adaptation to noisy Galvanic vestibular stimulation is associated with enhanced sensorimotor performance in novel vestibular environments

**DOI:** 10.3389/fnsys.2015.00088

**Published:** 2015-06-08

**Authors:** Steven T. Moore, Valentina Dilda, Tiffany R. Morris, Don A. Yungher, Hamish G. MacDougall

**Affiliations:** ^1^Human Aerospace Laboratory, Department of Neurology, Icahn School of Medicine at Mount SinaiNew York, NY, USA; ^2^School of Psychology, University of SydneySydney, NSW, Australia

**Keywords:** microgravity, GVS, dual adaptation, pre-habilitation, countermeasure

## Abstract

Performance on a visuomotor task in the presence of novel vestibular stimulation was assessed in nine healthy subjects. Four subjects had previously been adapted to 120 min exposure to noisy Galvanic vestibular stimulation (GVS) over 12 weekly sessions of 10 min; the remaining five subjects had never experienced GVS. Subjects were seated in a flight simulator and asked to null the roll motion of a visual bar presented on a screen using a joystick. Both the visual bar and the simulator cabin were moving in roll with a pseudorandom (sum of sines) waveform that were uncorrelated. The cross correlation coefficient, which ranges from 1 (identical waveforms) to 0 (unrelated waveforms), was calculated for the ideal (perfect nulling of bar motion) and actual joystick input waveform for each subject. The cross correlation coefficient for the GVS-adapted group (0.90 [SD 0.04]) was significantly higher (*t[8]* = 3.162; *p* = 0.013) than the control group (0.82 [SD 0.04]), suggesting that prior adaptation to GVS was associated with an enhanced ability to perform the visuomotor task in the presence of novel vestibular noise.

## Introduction

In-flight studies demonstrate a central reweighting of sensory input in microgravity, emphasizing vision and somatosensation to compensate for the lack of low-frequency gravitational input. On Earth, otolithic input encodes head angle (in the form of linear acceleration) with regard to gravity, which is centrally integrated with angular velocity input from the semicircular canals, vision, and proprioceptive information from neck receptors. For example, the roll vestibulo-ocular reflex, which uses this sensory input via direct and indirect pathways, is a broad frequency response that rotates the eyes in a manner compensatory for roll (lateral) head motion that tends to maintain retinal stability. When tilting the head in microgravity otolith input is absent; thus, input from vision, the vestibular end-organs and neck proprioception is in conflict in terms of the now maladapted terrestrial baseline state. Consequentially, the gain of the roll vestibulo-ocular reflex is diminished in microgravity by 20–30% (Clarke et al., 2000). However, gain recovers over the course of 6 months in space, approaching the pre-flight baseline. An explanation for this restoration of function is a central sensory reweighting that places increased emphasis on neck proprioception and vision during lateral head tilt in-flight to compensate for the lack of otolith input. Visual and perceptual responses showed a similar adaption (Young et al., 1996; Clément et al., 2001). Thus, astronauts become well adapted to the microgravity environment over a period of several weeks, up-weighting visual and somatosensory input where appropriate to compensate for the lack of low-frequency otolith input and unloading of stretch receptors throughout the body.

When returning to Earth (and presumably other planetary bodies) this microgravity-adapted state persists. Within hours of landing computerized dynamic posturography results from shuttle crewmembers demonstrated increased reliance on somatosensation and vision and down-weighting of vestibular input (Black et al., 1995), a state now maladapted to a gravitational environment. The gain of the roll vestibulo-ocular reflex described above, which had recovered to the preflight baseline after 6 months in space, dropped to early in-flight levels immediately post-flight, recuperating over 5–10 days (Clarke et al., 2000). The sensorimotor consequences for centrally-mediated processes utilizing low-frequency otolith input are well documented; impaired motion perception (Harm et al., 1999), postural imbalance when vision or proprioception is compromised (Paloski et al., 1999), and gait instability (Bloomberg et al., 1997). Diminished piloting ability has been observed even after short duration missions. Our review of the first 100 shuttle landings found that touchdown speed was outside of acceptable limits in 20 cases (Moore et al., 2008), the vast majority of which (19/20) were “hot” (above target and potentially damaging to the landing gear); the maximum allowable touchdown speed of 217 kn (main tires are rated at 225 kn max; NASA, 2000; Jenkins, 2001) was equaled or exceeded on six occasions (Moore et al., 2008). The two hardest touchdowns on record (STS-3 and STS-90) involved microgravity-induced spatial disorientation in the final stages of landing (Moore et al., 2008, 2011). Most studies report a post-landing recovery period of around 2 weeks for sensorimotor function (see Paloski et al., 2008), which likely reflects the process of central sensory reweighting to re-incorporate gravitational (otolith) input.

We have developed an analog of the effects of microgravity exposure on terrestrial sensorimotor performance utilizing bipolar bilateral Galvanic vestibular stimulation (GVS). Passing a small (5mA peak), low-frequency (<0.6 Hz) pseudorandom current waveform through external mastoidal electrodes activates neurons from both the otoliths and semicircular canals (Goldberg et al., 1984; Kim and Curthoys, 2004). The CNS interprets the resultant sum of vestibular afferents activated by GVS as a head tilt in the direction of the cathode (Fitzpatrick and Day, 2004), and generates small reflex responses towards the anode, consistent with a primarily otolith response (Dilda et al., 2014). Our studies have shown that anteroposterior stability (cerebellum; MacDougall et al., 2006), obstacle course navigation (cortex/cerebellum; Moore et al., 2006), and fine motor control (cortex/cerebellum; Moore et al., 2011), as well as short-term spatial memory (hippocampus), perspective taking and perception of motion (cortex; Moore et al., 2011; Dilda et al., 2012), are degraded by bilateral bipolar GVS. These findings suggest that imposing pseudorandom GVS “noise” on veridical vestibular input at the spike trigger zone negatively impacts upstream central functions that rely on integrated otolith, visual and somatosensory input. We have leveraged this destabilizing effect of GVS on sensorimotor function to replicate in healthy subjects the decrements in postural (MacDougall et al., 2006), locomotor (Moore et al., 2006), oculomotor (Moore et al., 2006), and piloting (Moore et al., 2011) performance observed in astronauts after spaceflight.

Veteran astronauts maintain critical elements of microgravity adaptation (dual adaptation), enabling faster switching between baseline and micro-gravitational states, and as a consequence, reduced sensorimotor impairment on subsequent flights (Davis et al., 1988; Bloomberg et al., 1997; Paloski et al., 1999). We propose a training regimen to establish dual-adaptation to novel vestibular environments prior to flight, utilizing GVS. In a recent study (Dilda et al., 2014) postural and locomotor performance in subjects undergoing 120-min cumulative GVS exposure over 12 weeks (10 min per week) was initially severely compromised, but recovered to baseline over a period of 7–8 weeks, and this recovery was maintained 6 months after adaptation (Dilda et al., 2014). In contrast, the roll vestibulo-ocular reflex response to GVS was not attenuated by repeated exposure. This suggests that GVS adaptation did not occur at the vestibular end-organs or involve changes in low-level (brainstem-mediated) vestibulo-ocular or vestibulo-spinal reflexes. Faced with unreliable vestibular input, the cerebellum reweighted sensory input to emphasize veridical extra-vestibular information, such as somatosensation, vision and visceral stretch receptors, to regain postural function. After a period of recovery subjects exhibited dual adaption and the ability to rapidly switch between the GVS and natural vestibular state for up to 6 months, analogous to veteran astronauts exhibiting dual adaptation to microgravity and terrestrial environments.

In this study we tested the hypothesis that prior adaptation to GVS was associated with enhanced sensorimotor performance during novel inertial (non-GVS) stimuli relative to GVS-naive control subjects. A visuomotor task was developed based on our studies of the difficulties encountered by astronaut pilots during shuttle landing (Moore et al., 2008, 2011). After extended exposure to microgravity astronauts are maladapted to gravitational levels encountered during approach and landing. Perception of motion is often impaired, with an exaggerated sense of tilt (the g-excess effect; Gillingham and Wolfe, 1985), persistence of surround motion when moving the head (Harm et al., 1999), and tumbling sensations (Moore et al., 2008). Pilots must reorder sensory input such that vision is weighted more heavily than vestibular input (to this end shuttles were fitted with a head-up display after the 6th mission of Columbia; Moore et al., 2008). We implemented a simple simulation of this scenario using a full-motion flight simulator developed for our NASA flight experiment (*NNX12AM25G—Assessment of operator proficiency following long-duration spaceflight*). Subjects were instructed to null the motion of the visual bar with a joystick (i.e., maintain it is as close to cabin vertical as possible) in the presence of the novel destabilizing roll vestibular input. Success in this task required the subject to attend to the visual signal while suppressing the conflicting vestibular input from simulator motion. Based on our GVS adaptation results (Dilda et al., 2014), we expected that GVS-trained subjects would be better able to accomplish this sensory reweighting.

## Methods

### Ethics Statement

Nine healthy subjects, 6 males/3 females, with a mean age of 26.1 years (SD 2.0), participated in this study. The Program for the Protection of Human Subjects (PPHS) at Icahn School of Medicine at Mount Sinai approved the experiments (study 07–0468), and subjects gave their written informed consent and were free to withdraw at any time.

### Galvanic Vestibular Stimulation (GVS) Training

The pseudorandom bilateral-bipolar Galvanic stimulus consisted of a sum-of-sines (0.16, 0.33, 0.43, 0.61 Hz) with peak amplitude of 5 mA (MacDougall et al., 2006; Moore et al., 2006) delivered by an optically-isolated constant current generator to the surface of the subject’s skin via leads and large (10 cm^2^) electrodes placed over the mastoid processes. Four of the subjects had previously undergone GVS training, with 12 weekly exposures of GVS (10 min per session) for a cumulative exposure of 120 min (Dilda et al., 2014). These GVS-adapted subjects were tested within 4 weeks of the final GVS training session.

### Visuomotor Task

**Figure 1:**
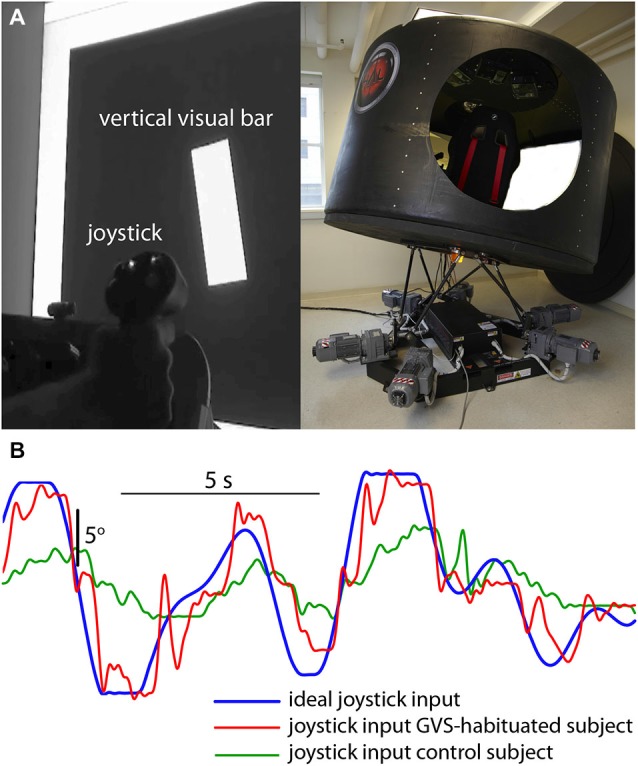
**Visuomotor performance in the presence of novel vestibular perturbation. (A)** Subjects were required to null the random roll motion of a vertical bar with a joystick, while the simulator cabin moved in roll with a conflicting pseudorandom motion. **(B)** Ideal input (blue) required to null the bar rotation, and joystick input from a GVS-adapted (red) and GVS-naive (green) subject.

Participants were seated in a cabin fixed to a six degree-of-freedom Stewart platform (V7, CKAS, Melbourne, Australia) and viewed a large display screen 1-m distant. The screen displayed a vertical bar that moved in roll in a pseudorandom manner up to a maximum of ±10° with respect to the cabin (screen) vertical (Figure 1A). Subjects had no visual cue of the outside environment. Simultaneously, the cabin was moved in the roll plane (max ±10°) with a different (conflicting) pseudorandom waveform to the visual perturbation (these waveforms were a zero-mean sum-of-sines [7 sinusoids varying between 0.14 and 1.0 Hz] similar to the post-flight motion-nulling experiment of Merfeld et al. (1996), and had not been previously experienced by either the control or GVS-adapted subjects). Subjects were instructed to null the motion of the visual bar with a joystick (i.e., maintain it is as close to cabin vertical as possible) in the presence of the novel destabilizing vestibular input. To compare subject performance the cross-correlation coefficient *r_xy_* was used to compare the actual joystick input waveform *x* with the ideal input *y* required to null the visual display motion (Figure 1B),

$$r_{xy=}\frac{c_{xy}}{\sqrt{c_{xx}c_{yy}}}$$

where *c_xy_* is the maximum amplitude of the cross-correlation between waveforms *x* and *y*, and *c_xx_* and *c_yy_* are the maximum values of the autocorrelation of *x* and *y*, respectively. The result, *r_xy_*, indicates the similarity of the two waveforms, with a range from 0 (independent waveforms) to 1 (identical waveforms; Li and Caldwell, 1999).

### Experimental Protocol

Subjects, four GVS-adapted (3 males, 1 females, mean age 26.1 years [SD 2.0]) and five GVS-naive controls (3 males, 2 females, mean age 26.0 years [SD 2.0]) were seated in the simulator cabin and asked to continuously null the rotation of the vertical bar displayed on the screen, such that it was aligned as closely as possible with the cabin “vertical” over 3 min. The cabin tilted unpredictably in roll throughout testing.

## Results

The correlation coefficient for the GVS-adapted group (0.90 [SD 0.04]) was significantly higher (*t[8]* = 3.162; *p* = 0.013) than the control group (0.82 [SD 0.04]), suggesting that prior adaptation to GVS was associated with an increased ability to perform the visuomotor task in the presence of novel vestibular noise.

## Discussion

The results of this study suggest that prior adaptation to a cumulative 120-minutes of noisy GVS was associated with enhanced visuomotor performance in the presence of a novel, non-GVS vestibular perturbation. GVS-trained subjects were significantly better than GVS-naive controls in nulling the roll motion of a visual bar during pseudorandom roll motion in a flight simulator. This result suggests that the benefits of adaptation to Galvanic stimulation, in which subjects successfully recovered full postural and locomotor function in the presence of GVS, were generalizable to novel vestibular environments.

The primary limitations of this study are the relatively limited number of subjects and the lack of a pre-GVS adaptation baseline for the visuomotor nulling task. It is possible, therefore, that the difference observed in visuomotor performance between the GVS-adapted and GVS-naive groups was due solely to an innate difference in visuomotor ability between the groups. However, we feel that this is unlikely. The subject groups were closely matched for age and gender (all were drawn from medical students), the SD of mean group performance was low (4%), and the difference between group means was 2 SD. However, future double-blind sham-controlled studies are needed to fully assess the validity of GVS adaptation training.

Adaptation to GVS occurred centrally, with full postural and locomotor recovery but no change in the vestibulo-ocular (brainstem mediated) reflex response to GVS (Dilda et al., 2014). In- and post-flight results suggest an analogous central adaptation to the lack of gravity (Young et al., 1996; Clarke et al., 2000; Clément et al., 2001). In both cases data suggests a reweighting of sensory input at the level of the cerebellum, emphasizing extra-vestibular information to compensate for degraded low-frequency otolith input. Moreover, both astronauts and GVS-trained subjects exhibit dual adaptation, the ability to switch between a perturbed (GVS, microgravity) and nominal vestibular state whilst maintaining sensorimotor function. Acquisition of dual-adaptation to spaceflight occurs in a linear fashion, requiring crewmembers to experience actual missions to develop the ability to toggle between baseline and microgravity-adapted states. Ideally, pre-flight training would pre-adapt astronauts to microgravity, fast-tracking the establishment of dual-adaptation and the ability to rapidly switch between states in-flight. The results of the current study demonstrate that the beneficial aspects of adaptation to GVS generalize to novel vestibular perturbations. Coupled with the findings that GVS training facilitates dual adaptation and that the benefits persists at least 6-months after exposure (Dilda et al., 2014), we propose that pre-adaptation to Galvanic vestibular stimulation may improve sensorimotor performance in the novel inertial environment of spaceflight. In a similar manner, GVS adaptation may be useful in other aerospace or maritime applications, and for vestibular “pre-habilitation” (Magnusson et al., 2011), in which patients with planned unilateral lesions adapt to repeated provocative vestibular stimuli prior to intervention to minimize the impact of a post-lesion imbalance in vestibular tone.

## Conflict of Interest Statement

The authors declare that the research was conducted in the absence of any commercial or financial relationships that could be construed as a potential conflict of interest.
